# Supplementary material for: Unveiling the double-peak structure of quantum oscillations in the specific heat
Source: Nat Commun. 2023 Nov 8;14:7006. doi: 10.1038/s41467-023-42730-4 (PMC10632398; doi:10.1038/s41467-023-42730-4)
Supplement: Supplementary file 1 — Supplementary information [file 41467_2023_42730_MOESM1_ESM.pdf]

Supplementary information for: Unveiling the double-peak structure of quantum oscillations in the specific heat

Zhuo Yang,[1,*] Benoît Fauqué,[2] Toshihiro Nomura,[1] Takashi Shitaokoshi,[1] Sunghoon Kim,[3] Debanjan Chowdhury,[3] Zuzana Pribulová,[4] Jozef Kačmarčík,[4] Alexandre Pourret,[5] Georg Knebel,[5] Dai Aoki,[6] Thierry Klein,[7] Duncan K. Maude,[8] Christophe Marcenat,[5] and Yoshimitsu Kohama[1]

[1]*Institute for Solid State Physics, The University of Tokyo, Kashiwa, Chiba, 277-8581, Japan*

[2]*JEIP, USR 3573 CNRS, Collège de France, PSL Research University, 11, Place Marcelin Berthelot, 75231 Paris Cedex 05, France*

[3]*Department of Physics, Cornell University, Ithaca NY 14853, USA*

[4]*Centre of Low Temperature Physics, Institute of Experimental Physics, Slovak Academy of Sciences, Watsonova 47, SK-04001 Košice, Slovakia*

[5]*Univ. Grenoble Alpes, CEA, Grenoble INP, IRIG, Pheliqs, 38000 Grenoble, France*

[6]*Institute for Materials Research, Tohoku University, Oarai, Ibaraki 311-1313, Japan*

[7]*Univ. Grenoble Alpes, CNRS, Institut Néel, 38000 Grenoble France*

[8]*Laboratoire National des Champs Magnétiques Intenses, CNRS-UGA-UPS-INSA, 143 avenue de Rangueil, 31400 Toulouse, France*

Supplementary Note 1: Deviation from LK theory in quantum oscillation of specific heat in graphite

In this section, we briefly introduce the expression of quantum oscillations in specific heat predicted in LK theory, and compare it with the double-peak structure observed in experimental results. The oscillatory component of specific heat due to Landau level quantization of the orbits is given by the extended Lifshitz-Kosevich formula [1],

$$\Delta C_{el}(T,B) = AT\sum_{p=1}^{\infty} R_D J_0(4\pi p\frac{t_w}{\hbar\omega_c})\cos(2\pi p(\frac{\mu}{\hbar\omega_c}-\frac{1}{2}))\phi(z), \quad (1)$$

where A is a constant, $R_D = exp(-2\pi^2 pk_BT_D/(\hbar\omega_c))$ is the Dingle term with dingle temperature T_D, J_0 is a Bessel function of the first kind, t_w is the c-axis hopping energy, μ is the chemical potential, $\hbar\omega_c = \hbar eB/m^*$ is cycltron energy, $\phi(z) = z(2\cosh(z)/\sinh^2(z) - z(1 + \cosh^2(z)/\sinh^3(z)))$ with $z = 2\pi^2 pk_BT/(\hbar\omega_c)$. It is important to note that $\phi(z)$ changes sign at $z = 1.6$ which induces a "π-phase shift" for $\Delta C_{el}(T,B)$. When T and B satisfy the condition of $z = 1.6$, the amplitude of $\Delta C_{el}(T,B)$ drops to zero, which can be used to determine the effective mass m^* [2–4]. This kind of π phase shift in specific heat quantum oscillations has been reported in the organic superconductor (BEDT-TTF)$_2$Cu(NCS)$_2$ [3] and unusual interplay between superconductivity and field-induced charge order in $YBa_2Cu_3O_y$ [2, 4, 5].

The π-phase shift of the oscillatory components is indeed observed in graphite at low magnetic field ($B < 0.9$ T) with Landau indexes $N > 6$, which is consistent with the prediction of the extended LK formula [6]. The quantum oscillation of specific heat in graphite is a mixture of oscillatory component from both electron and hole pockets, which make it difficult to find the zero amplitude of $\Delta C_{el}(T,B)$. Therefore, we first calculated the temperature-field boundary of the π-phase shift based on the known effective mass for electron and holes in graphite, and compare it with our experimental data. Supplementary Fig. 1a shows the calculated boundary, T_b, for the π-phase shift ($z_{e/h} = 1.6$) using $0.056\,m_e$ and $0.067\,m_e$ as the electron and hole effective mass [7]. The π-phase shift occurs around 1-1.5 K in the field range from 0.55 T to 0.85 T. Supplementary Fig. 1b shows the field sweep of C_{el}/T taken in the temperature range of 0.3 - 2 K. The Landau index for each oscillatory component are marked. It is clear that the C_{el}/T has a π-phase shift between the 0.5 K and 1.5 K data, which is consistent with our calculation in Supplementary Fig. 1a.

* zhuo.yang@issp.u-tokyo.ac.jp

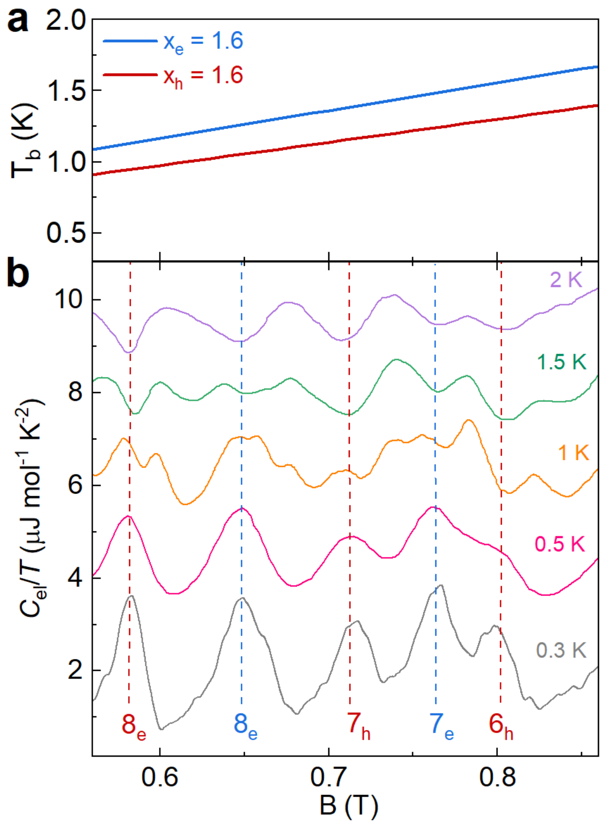

Supplementary Figure 1. **a** Calculated boundary for the π-phase shift of electron and hole pockets. **b** Oscillatory component of C_{el}/T with high Landau index levels at indicated temperature. The dashed lines demonstrate the π-phase shift between 0.5 K and 1.5 K. The curves are vertically offset for clarity.

It is clear that the extended LK theory predicts a single peak feature in C_{el}/T when an individual Landau level passes over the Fermi level, which has been widely used in the literature [2–5]. Together with the double-peak feature reported in this study, we conclude that the quantum oscillations of $C_{\rm el}/T$ can appear as either single or double-peak features, depending on the width of Landau level, temperature and dE/dB. This suggests that care must be paid when we are trying to extract the frequency of quantum oscillations, since the frequency of the oscillations can be two times higher than the real case in the double-peak structure region.

Supplementary Note 2: Electronic specific heat of graphite

The electronic specific heat C_{el} was obtained by subtracting the phonon contribution C_{ph} from the total specific heat of the specimen C_{tot}. At zero field and low temperature, the C_{tot}/T is linearly dependent on T^2, and can be well described by [8],

$$C_{tot}/T = \gamma + \beta T^2, \tag{2}$$

where γ is Sommerfeld coefficient, βT^2 stands for the acoustic phononic contribution to the specific heat. From the fitting of zero field specific heat data, we found $\gamma = 20\pm3\ \mu\mathrm{J}\cdot\mathrm{K}^{-2}\cdot\mathrm{mol}^{-1}$ and $\beta = 28\pm3\ \mu\mathrm{J}\cdot\mathrm{K}^{-4}\cdot\mathrm{mol}^{-1}$ [9]. Since the phononic contribution in graphite is field independent, it is reasonable to subtract the phononic contribution for all the field range using the β value obtained at $B = 0\,\mathrm{T}$.

Supplementary Note 3: Verification of intrinsic effect

In order to verify that the double-peak structure in $C_{\rm el}/T$ is an intrinsic effect, we checked the reproducibility for different samples, the expected quasi-2D angle-dependence, and the reproducibility of up and down field sweeps.

Supplementary Note 3.1: Reproducibility of double-peak structure for different samples

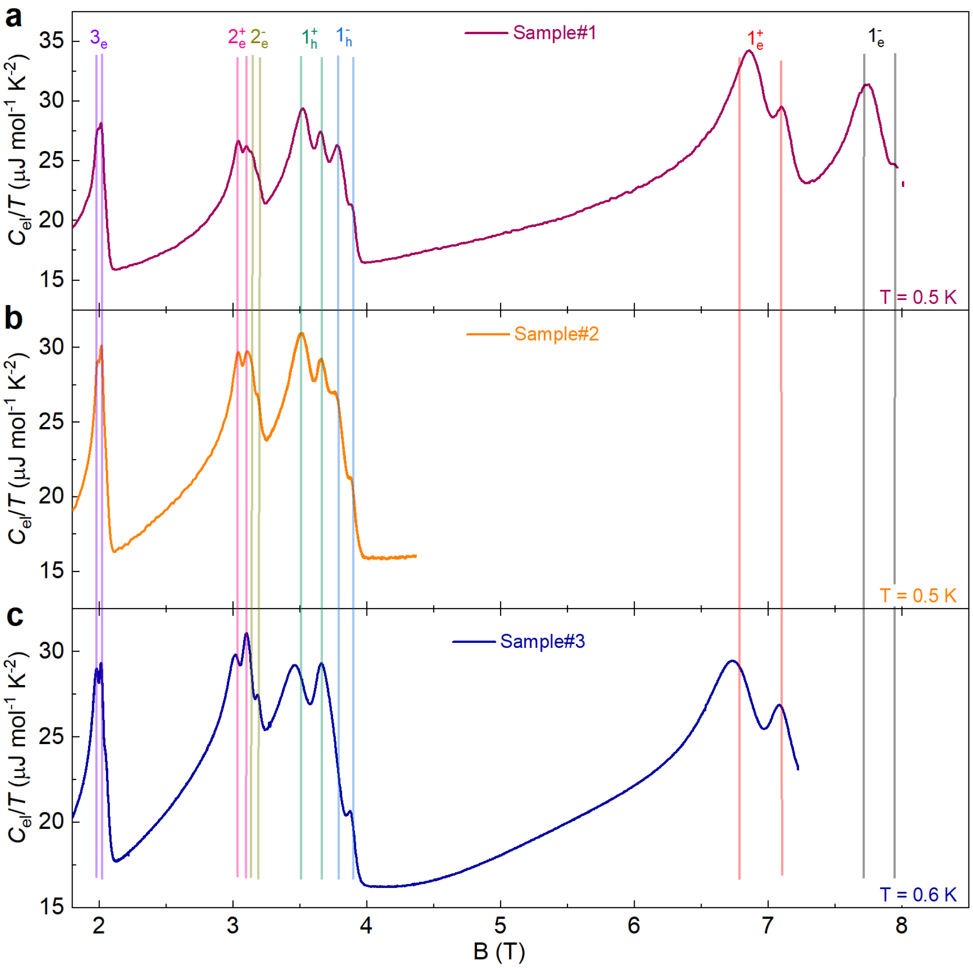

Supplementary Figure 2. **a - c** Field sweep electronic specific heat divided by temperature $C_{\rm el}/T$ for Sample#1 - Sample#3 as a function of magnetic field. Sample#1 and Sample#2 were measured at $T = 0.5\,{\rm K}$. Sample#3 was measured at $T = 0.6\,{\rm K}$.

Supplementary Fig. 2a-c shows the field-sweep electronic specific heat $C_{\rm el}/T$ at $T \simeq 0.5\,{\rm K}$

on three different natural graphite (Sample#1 - Sample#3). Vertical lines are guides to the eye, indicating the consistency between different sample. All three samples exhibit clear double-peak structures for each single spin-split Landau level, suggesting a good reproducibility of the double-peak structure.

Supplementary Note 3.2: Angle-dependence of double-peak structure

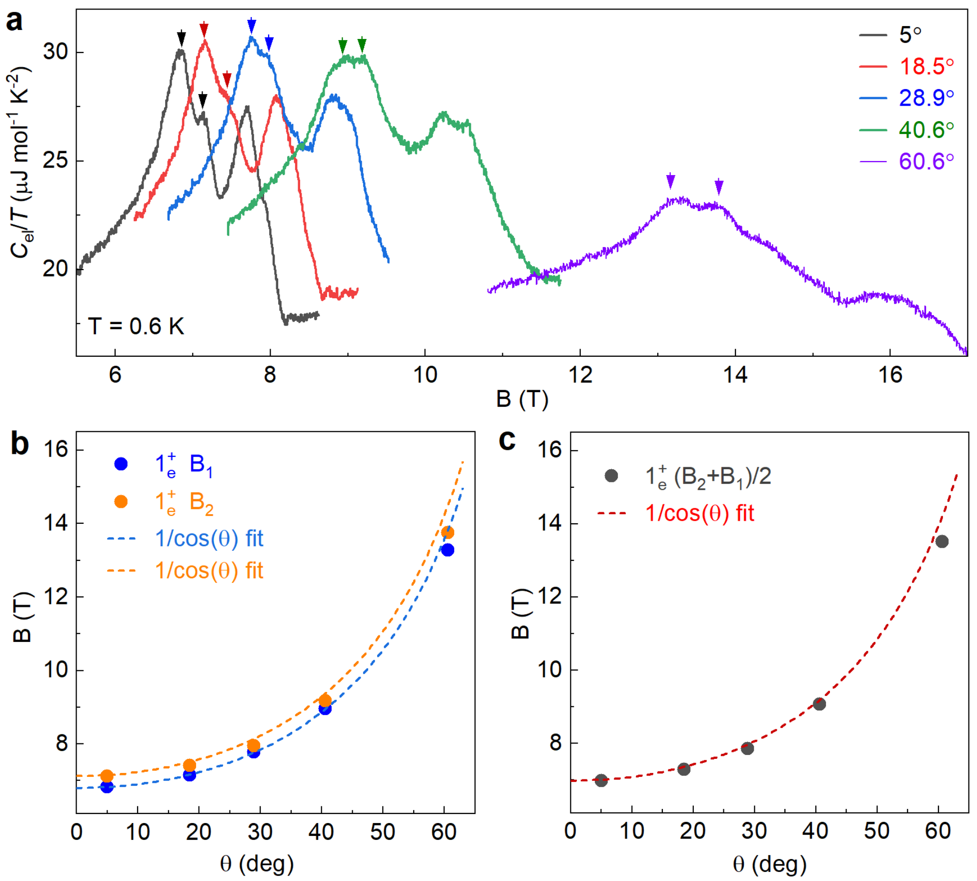

Supplementary Figure 3. **a** Field sweep electronic specific heat divided by temperature C_{el}/T of Sample#2 measured at $T = 0.6\,\mathrm{K}$ and indicated angle. **b** Angle-dependent peak position B_1, B_2 for 1_e^+ level. **c** Angle-dependent $(B_1 + B_2)/2$ for 1_e^+ level.

Graphite has a closed 3D Fermi surface, nevertheless, it shows a quasi-2D behavior in tilted magnetic fields, at least for tilt angles $\theta \leq 70°$ [10]. To verify the intrinsic nature of the double peak structure, we have performed angle-dependent field sweep specific heat measurement on Sample#2 at $T = 0.6\,\mathrm{K}$, as seen in Supplementary Fig. 3a. In this measure-

ment, we focused on the double-peak structure for the 1_e^+ level, where the splitting of double peaks are most clearly resolved. In Supplementary Fig. 3b, we show the magnetic positions of the double peaks B_1 B_2 of 1_e^+ level as a function of angle θ. Dashed lines are the fitting using $1/\cos(\theta)$. The consistency between fitting and data points suggests that double-peaks follows the quasi-2D rule in magnetic field. The center of double-peaks $(B_1 + B_2)/2$ also follows the quasi-2D rule, as shown in Supplementary Fig. 3c.

Supplementary Note 3.3: Reproducibility between up and down magnetic field sweeps

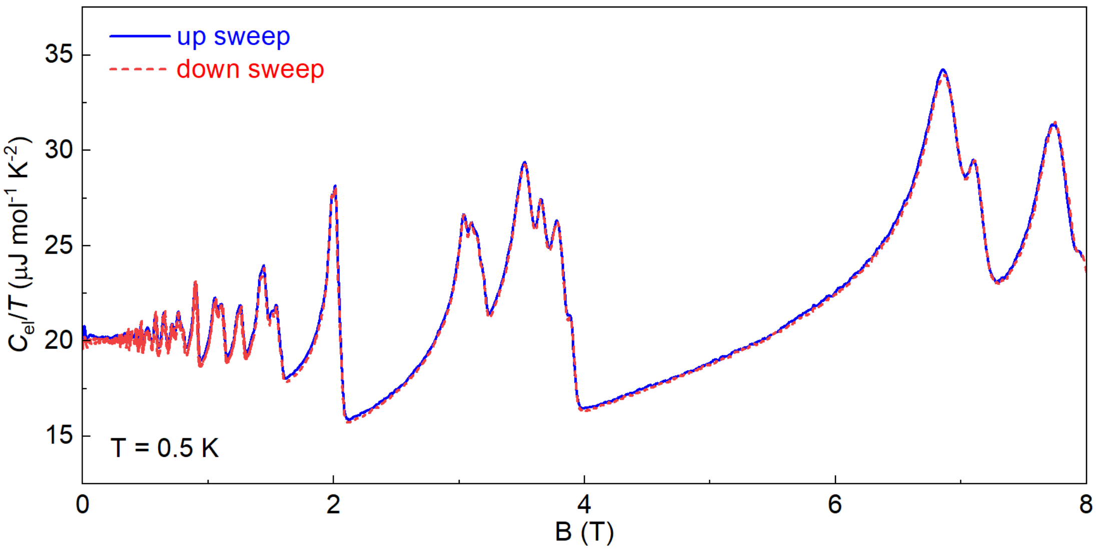

Supplementary Figure 4. Field sweep electronic specific heat divided by temperature C_{el}/T of Sample#1 measured at $T = 0.5$ K. Red dashed and blue solid lines represent C_{el}/T obtained in the up sweep and down sweep of the magnetic field, respectively.

In general, the extrinsic effect originated from the experimental setup will induce discrepancy between the up sweep and down sweep field data. In Supplementary Fig. 4, we show the C_{el}/T on Sample#1 measured at up sweep (blue solid line) and down sweep (red dashed line) of the magnetic field. The two curves are almost identical, notably there is no hysteresis, therefore, we conclude that the double-peak structures are not extrinsic effect from the experimental setup.

Supplementary Note 4: Caculation of Landau level and Fermi energy shift within SWM-model

In this section, we show how the Landau levels and the movement of Fermi energy were calculated within SWM-model. Graphite is a semi-metal with the carriers occupying a small region along the $H-K-H$ edge of the hexagonal Brillouin zone. The SWM Hamiltonian [11, 12] with its seven tight binding parameters $\gamma_0, ..., \gamma_5, \Delta$ provides a remarkably accurate description of the band structure of graphite [13, 14]. In a magnetic field, when trigonal warping is included ($\gamma_3 \neq 0$) levels with orbital quantum number N couple to levels with orbital quantum number $N+3$ and the Hamiltonian has infinite order. Nevertheless, the infinite matrix can be truncated and numerically diagonalized, as the eigen-values converge rapidly [15].

Supplementary Table 1 shows the value of SWM parameters that used in this study, taken from the SWM parameter set optimized to fit de Haas-van Alphen measurements in natural graphite[10]. They vary very little from the published values in other reports, *e.g.* [7, 14, 16].

Supplementary Table 1. Summary of the parameters used in the SWM tight binding Hamiltonian which are taken from [10]. The parameters $\gamma_1, ...\gamma_5, \Delta$ are given in units of eV.

γ_0	γ_1	γ_2	γ_3	γ_4	γ_5	Δ	g_s	E_F (meV)
3.15	0.375	-0.0243	0.443	0.07	0.05	-0.002	2.5	-26.1

Under magnetic field, the band structure of graphite become quasi-one-dimensional, depending only on the wave vector along z-direction, which greatly simplifies our calculation. The calculated band structure along k_z direction at $B = 2.5\,\mathrm{T}$ using our SWM parameters is shown in Supplementary Fig. 5. In the presence of magnetic field, the bands split into Landau bands, as indicated by black solid curves. In this study, we focus on the specific heat feature for the crossing of singularity DOS and the Fermi energy. The singularity DOS locates at the local extreme of a given Landau band [17], which can be found by the following relation,

$$\frac{d(E_N)}{dk_z}(B) = 0, \tag{3}$$

where E_N is the Landau band energy with index N. The local minima of electron Landau band and maxima of hole Landau band are marked as blue and red dots in Supplemen-

tary Fig. 5. Then, by calculating the energy of local extreme at various magnetic field, we obtained the Landau level for electrons and holes in Manuscript Fig. 1d.

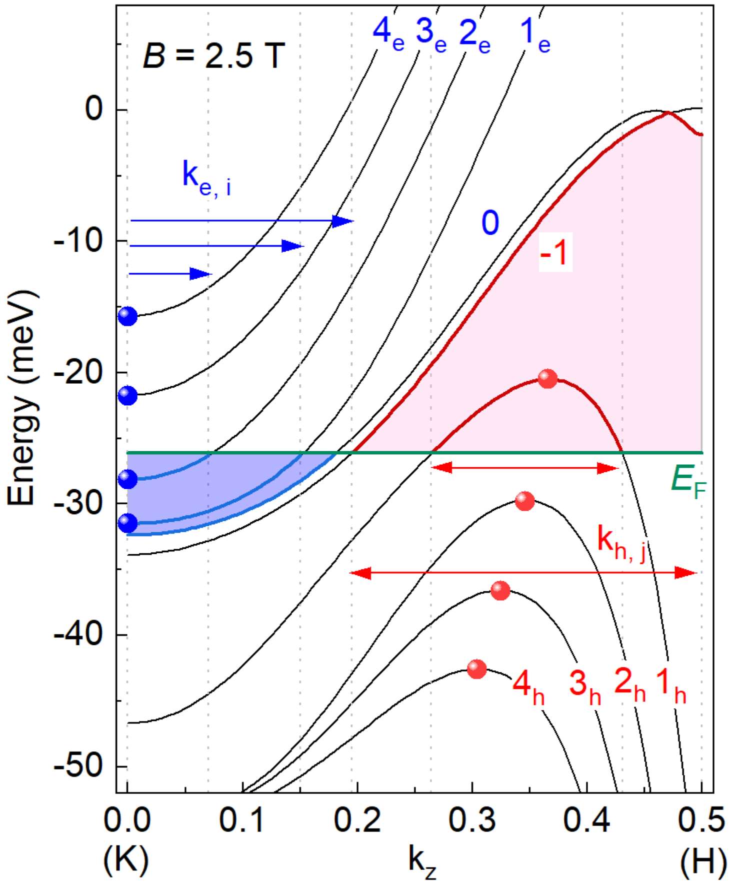

Supplementary Figure 5. Band structure of graphite at $B = 2.5\,\mathrm{T}$ calculated from SWM-model. Blue and red transparent area represent the electron and hole pockets. The local extrema for Landau levels are marked with blue (electrons) and red (holes) dots.

It has been shown in the transport measurement that the movement of Fermi energy of graphite is negligibly small at $B < 2\,\mathrm{T}$, but becomes significant at $B > 2\,\mathrm{T}$ [14]. The fundamental principle behind this phenomenon is the "charge neutrality" condition, that is, the difference of the electron and hole concentration should be a constant value,

$$n_e - n_h = n_0, \tag{4}$$

where n_e and n_h are the electron and hole concentration, n_0 is a constant value representing the difference. Considering the degeneracy of the Landau bands, the electron and hole

concentrations are given by [17],

$$n_e = B \cdot \sum_i k_{e,i} \quad and \quad n_h = B \cdot \sum_j k_{h,j}, \tag{5}$$

here, $k_{e,i}$ and $k_{h,j}$ are the k_z distance for the occupied i-th electron and j-th hole Landau bands, as shown by blue and red arrows in Supplementary Fig. 5. At low magnetic field, many Landau bands are occupied, adding electrons or holes does not require significant changes of the Fermi level to fulfill the $n_e - n_h = n_0$ condition.While at high magnetic field, when only one or two Landau bands are occupied, the Fermi energy has to shift to fulfill this condition.

Supplementary Note 5: Landau level broadening Γ_q and quantum lifetime τ_q in graphite

In Supplementary Table 2, we summarize the quantum life times τ_q and Landau level broadening $\Gamma_q = \hbar/\tau_q$ estimated from the magnetic field for the onset of Shubnikov-de Haas oscillations ($\omega_c \tau_q = 1$) for both natural graphite (NG) and highly orientated pyrolytic graphite (HOPG) at mK temperatures [18]. The Γ estimated from the double-peak structure in C_{el}/T (0.18 - 0.21 meV) is very close to the Landau level broadening Γ_q for NG, but much smaller than the broadening Γ_q in HOPG. Note that our C_{el}/T measurements were performed on natural graphite. The consistency between the Γ extracted from C_{el}/T versus B and the Γ_q extracted from the onset of the Shubnikov-de Haas oscillations lends further support to our model.

Supplementary Table 2. Summary of quantum lifetime τ_q and Landau level broadening Γ_q extracted from onset of Shubnikov-de Haas oscillations for both NG and HOPG [18].

	NG		HOPG		
	electron	hole	electron	hole	unit
τ_q	4.6	3.3	1.2	0.9	ps
Γ_q	0.143	0.199	0.549	0.731	meV

Supplementary Note 6: Exact form for various thermodynamic and transport probes

The exact forms of the charge conductance (G), Magnetization (M), Entropy (S, note $S \propto 1/T$ *i.e.* the MCE effect), thermopower(L), specific heat (C) and thermal conductance (K), for example when a single DOS peak $D(E)$ passes through the Fermi energy, are given by [8, 19, 20],

$$G = 2\frac{e^2}{\hbar}\int_{-\infty}^{\infty} D(E)\left(-\frac{dF(x)}{dx}\right)dx, \tag{6}$$

$$M = \mu_B^2 B\int_{-\infty}^{\infty} D(E)\left(-\frac{dF(x)}{dx}\right)dx, \tag{7}$$

$$S = k_B^2\int_{-\infty}^{\infty} D(E)(-F(x)\ln(F(x)) - (1-F(x))\ln(1-F(x)))dx, \tag{8}$$

$$L = 2\frac{e^2}{\hbar}\frac{k_B}{e}\int_{-\infty}^{\infty} D(E)\left(-x\frac{dF(x)}{dx}\right)dx, \tag{9}$$

$$C/T = k_B^2\int_{-\infty}^{\infty} D(E)\left(-x^2\frac{dF(x)}{dx}\right)dx, \tag{10}$$

$$K/T = 2\frac{e^2}{\hbar}(\frac{k_B}{e})^2\int_{-\infty}^{\infty} D(E)\left(-x^2\frac{dF(x)}{dx}\right)dx, \tag{11}$$

Here $F(x) = (1 + e^x)^{-1}$ is the Fermi-Dirac distribution function, and the dimensionless parameter $x = E/k_BT$, where the energy E is measured with respect to the Fermi energy. Note, with the exception of the entropy, the thermodynamic and transport probes all depend on a kernel function of the form $-x^n dF/dx$ with $n = 0, 1, 2$. It is the value of n which decides the response of a given thermodynamic or transport probe when a DOS singularity crosses the Fermi energy. Note that, as discussed below, the derivative with respect to temperature of the entropy dS/dT does depend on the kernel function $-x^n dF/dx$ with $n = 2$.

Supplementary Note 7: Calculated entropy near the crossing point of a Landau level and the Fermi energy

In this section, we show the calculated entropy S in the vicinity of a crossing point of a Landau level with the Fermi energy in order to fully understand the magneto-caloric effect (MCE) results in the manuscript. A single Landau level DOS was constructed using Manuscript Eq. (2) with parameters listed in Supplementary Fig. 6a. The crossing field B_0 was set to 5 T. The entropy S_{el} for Fermionic quasiparticles is given by [21],

$$S_{el} = k_B^2 \int_{-\infty}^{\infty} D(E)(-F(x)\ln(F(x)) - (1-F(x))\ln(1-F(x)))dx, \tag{12}$$

where $F(x) = 1/(1+e^x)$, $x = E/k_BT$ and k_B is the Boltzmann constant. Taking a temperature derivative of Supplementary Eq. (12) results in,

$$\frac{dS_{el}}{dT} = k_B^2 \int_{-\infty}^{\infty} D(E)x^2(-\frac{dF(x)}{dx})dx = C_{el}/T, \tag{13}$$

which is exactly the expression of the specific heat divided by temperature.

S_{el} involves integral of the Landau level DOS and a term $-F(x)\ln(F(x)) - (1 - F(x))\ln(1-F(x))$. As shown in Supplementary Fig. 6a, this term exhibits a single-peak feature, in contrast to the double-peak feature that originated from $x^2(-dF(x)/dx)$ in C_{el}/T. The calculated S at various temperature is shown in Supplementary Fig. 6b. It is clear that S shows a single peak structure when the Landau level crosses through the Fermi energy. However, the differential entropy $dS_{el}/dT \propto C_{el}/T$ is expected to show the double-peak structure. In Supplementary Fig. 6c we plot the calculated differential entropy, $(S(2\text{K}) - S(1\text{K}))/1\text{K}$, and the expected double-peak feature is indeed present.

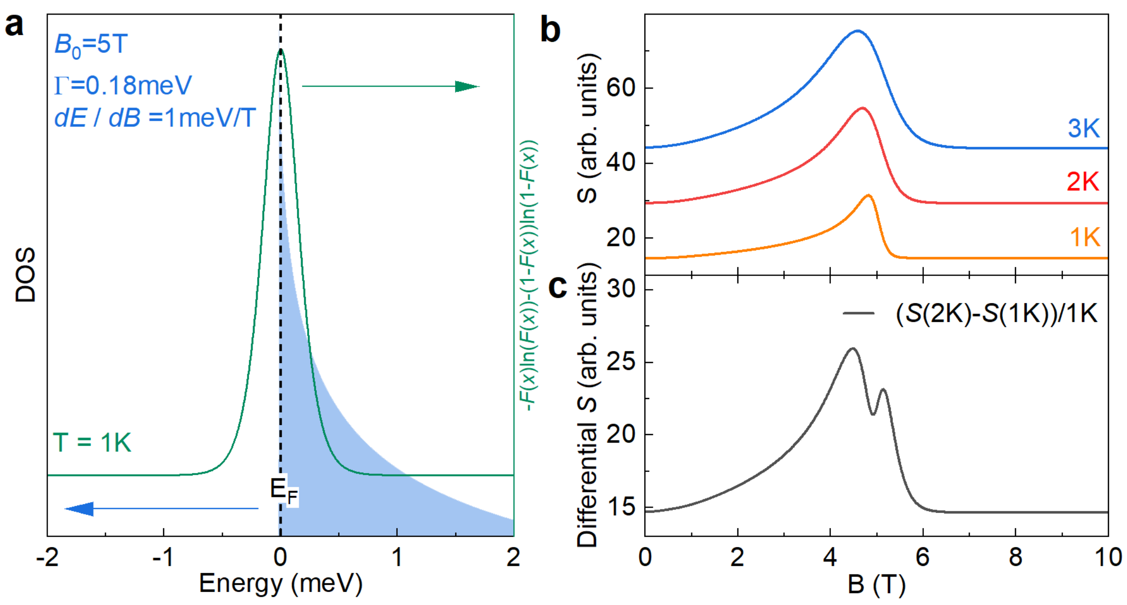

Supplementary Figure 6. **a** Constructed Landau level DOS that used to calculate the entropy. **b** Calculated entropy S ($\propto 1/T$ in MCE) for different temperatures in the vicinity of the crossing of Landau level with the Fermi energy. No double-peak structure is observed in the calculated entropy, in agreement with the absence of double-peak structure in MCE. **c** Calculated differential entropy in the vicinity of Landau level with Fermi energy. The double-peak structure is observed in the calculated differential entropy.

Supplementary Note 8: Maxima in the kernel function $-x^2dF(x)/dx$ of specific heat

In the manuscript we state without justification that the maxima in $-x^2dF(x)/dx$ occur at $x = \pm 2.4$. This value was determined by manually reading the peak position from a plot of the function. Here, for completeness, we attempt to derive this result using calculus. We define the Fermi-Dirac function $y = (1 + e^x)^{-1}$. Using the chain rule we can calculate the derivatives dy/dx and d^2y/dx^2. We define the kernel function $z = -x^2dy/dx$. We find the maxima in the kernel function by looking for zeros in the first derivative,

$$\frac{dz}{dx} = -2x\frac{dy}{dx} - x^2\frac{d^2y}{dx^2} = xy^2e^x(2 - 2xye^x + x), \tag{14}$$

The maxima in z correspond to the roots of the function

$$0 = (2 - 2xye^x + x), \tag{15}$$

Multiply both sides by y^{-1} and substituting $(1 + e^x)$ for y^{-1} we obtain after simplification,

$$0 = (2 + x) + (2 - x)e^x, \tag{16}$$

Despite the apparent simplicity of this function we failed to find an analytical solution for the roots. Instead we used Newton's method, to find the roots of a function f(x), using successive approximations, $x_{n+1} = x_n - f(x_n)/f'(x_n)$, using the initial guess $x_0 = \pm 2$. This method converges rapidly to give the roots $x = \pm 2.399357280515468$, justifying *a posteriori*, our approximation $x = \pm 2.4$.

Supplementary Note 9: Thermopower in graphite

As mentioned in the main text, the negative and positive peaks in the thermopower have been experimentally observed. In this section, we demonstrate this feature using the thermopower data of graphite in the literature [22, 23]. As a step forward, we predict the splitting of the negative and positive peaks of thermopower to be $\simeq 3.09 k_B T$ based on the kernel term.

Supplementary Note 9.1: Negative and positive peaks in thermopower of graphite

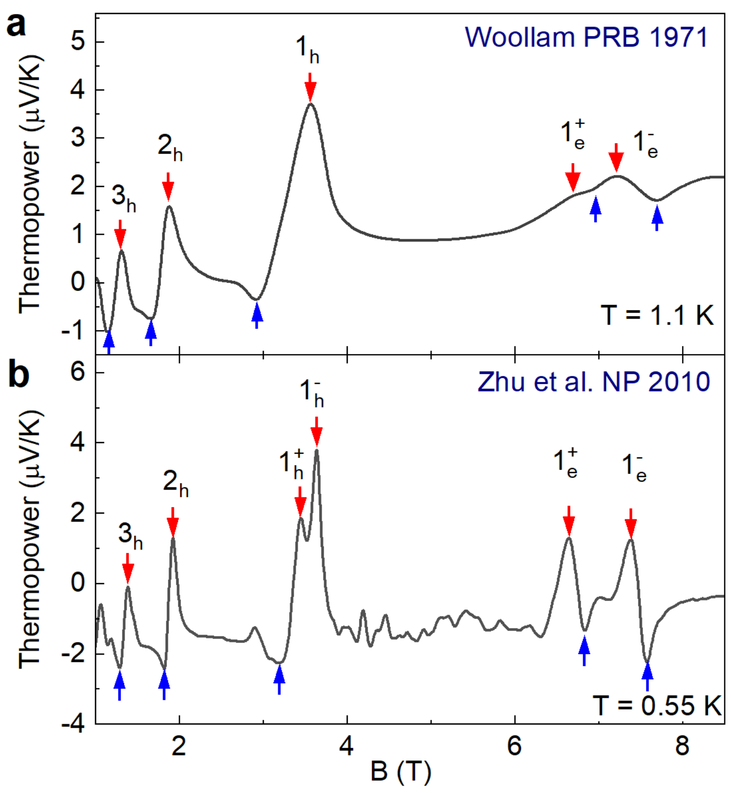

Supplementary Figure 7. Thermopower as a function of magnetic field for graphite. The experimental data were digitalized from ref [22] **a** and ref [23] **b**. The blue and red arrows indicate the negative and postive peak position in the thermopower for a single DOS peak passing over the Fermi level.

Supplementary Fig. 7 shows the thermopower versus magnetic field for graphite. The experimental data were digitalized from ref [22, 23]. As seen in Supplementary Fig. 7, the

thermopower shows a negative and a positive peak for each Landau level passing over the Fermi energy, as marked by blue and red arrows.

It is important to note that this negative/positive peak feature are opposite for the electrons and holes. Namely, the positive peak locates at lower magnetic field position for the electrons, but locates at higher magnetic field position for the holes.

Supplementary Note 9.2: Predictions for thermopower

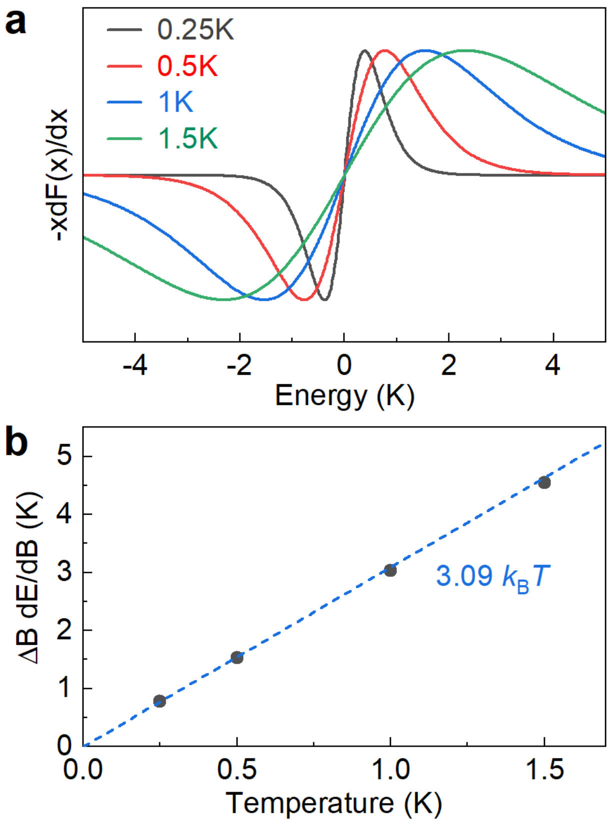

Supplementary Figure 8. **a** The kernel term for thermopower $-xdF(x)/dx$ in the vicinity of the Fermi energy calculated at different temperatures. **b** The dashed line shows the theoretical $3.09k_BT$ splitting between the maximum and minimum of the kernel function $-xdF(x)/dx$. The symbols are the splitting manually read off from the plotted $-xdF(x)/dx$ in panel **a**.

The kernel function for specific heat and thermal transport x^2dF/dx, with $x = E/k_BT$, is an even function of energy around the Fermi energy, with maxima at $x = \pm 4.8$. With the exception of thermopower, other thermodynamic probes have a kernel function with a single

maximum at $x = 0$. The kernel function for thermopower $-xdF/dx$ is an odd function of energy around the Fermi energy, as can be seen in Supplementary Fig. 8.

We define the kernel function $z = -xdy/dx$ with the Fermi-Dirac distribution function $y = (1 + e^x)^{-1}$. We find the maximum and minimum in the kernel function by looking for zeros in the first derivative,

$$\frac{dz}{dx} = -\frac{dy}{dx} - x\frac{d^2y}{dx^2} = y^3 e^x ((1 + x) + (1 - x)e^x), \tag{17}$$

The maximum and minimum in z correspond to the roots of the function

$$0 = (1 + x) + (1 - x)e^x. \tag{18}$$

Using Newton's method the roots occur at $x = \pm 1.543404638418208$, so that the expected splitting of the maximum and minimum in thermopower, when a DOS singularity crosses the Fermi energy is $\simeq 3.09 k_B T$.

Supplementary Note 10: Deviation from the expected $4.8k_BT$ double-peak structure splitting in specific heat due to the asymmetry of the Landau level DOS

The simple picture, in which maxima in $C_{el}/T \propto \int D(E)(-x^2dF(x)/dx)$ occur when the DOS peak lies at the centre of the maxima in kernel term $-x^2dF(x)/dx$ (located at $x = \pm 2.4$, $E = xk_BT$), has to be exact provided the DOS peak is symmetric (e.g. cusp-like DOS in Lifshitz transition). We validated this hypothesis by calculating the overlap integral versus energy, for different temperatures and width Γ, as the DOS peak passes through the Fermi energy. In Supplementary Fig. 9a, we plot the double-peak structure splitting $\Delta B\, dE/dB$ in the simulated C_{el}/T using a symmetric DOS peak, versus temperature. The dashed line is the expected variation if the splitting in C_{el}/T exactly mimics the $4.8k_BT$ splitting of $-x^2dF(x)/dx$. In the inset of Fig.9 (a), we plot the splitting in the calculated C_{el}/T as a function of the Landau level width Γ. This is simply the slope of the ΔE versus T plots in the main panel. As can be seen, the splitting in C_{el}/T for a symmetric DOS peak are in good agreement with expected $4.8k_BT$ regardless of the temperatures and the width Γ.

However, this is not exactly the case for a highly asymmetric DOS peak (e.g. DOS in Landau levels). The large asymmetric tail, on the high energy side of the 'singularity' ($D(E) = \beta/(1+\sqrt{(E-E_0)/\Gamma})$) causes the peaks in C_{el}/T to shift away from this condition. Although both peaks shift in the same direction (see Supplementary Fig. 10e), the shift of the peaks is not identical due to the asymmetric shape of DOS. For this reason, we performed similar simulation using a highly asymmetric DOS peak to evaluate how important are the deviations of the splitting in C_{el}/T from the $4.8k_BT$ splitting of the maxima in $-x^2dF(x)/dx$. In Supplementary Fig. 9b we plot the double-peak structure splitting in the simulated C_{el}/T using an asymmetric DOS peak, versus temperature. As can be seen, the splitting in C_{el}/T is larger than $4.8k_BT$ and the correction is of the order of 20% for $\Gamma = 0.2$ meV. When using the splitting in C_{el}/T to determine for example the electronic g-factors using the coincidence method, it is important to use the splitting which corresponds to the correct width of the Landau level, which can be extracted from the fit to the experimental C_{el}/T versus B data.

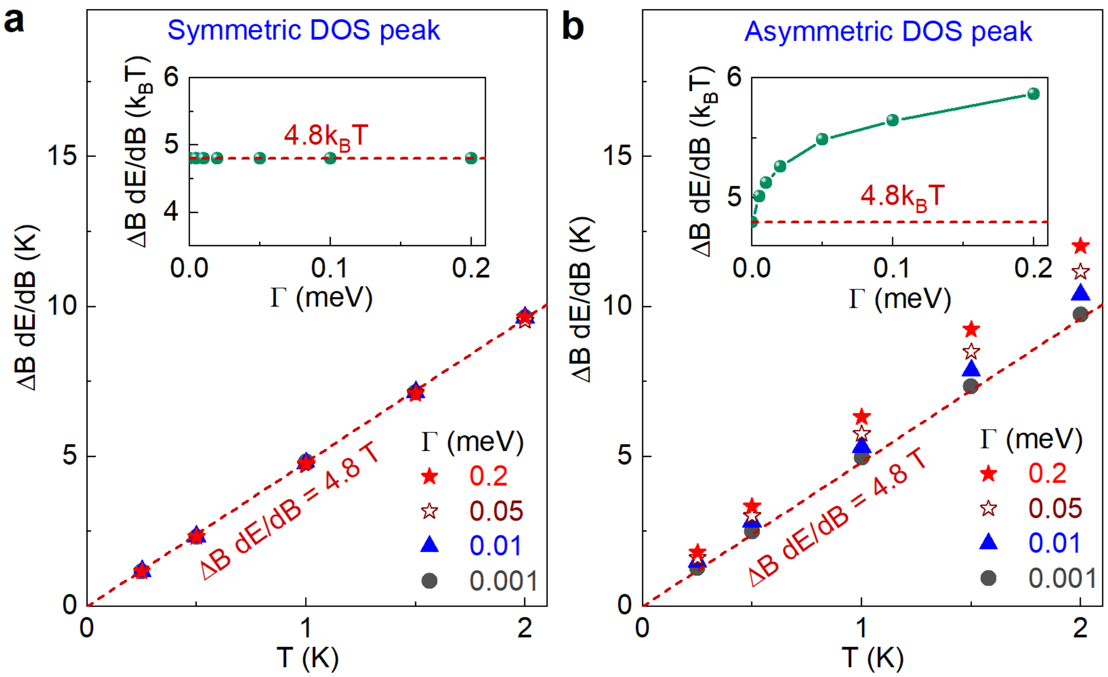

Supplementary Figure 9. Calculated double-peak structure splitting in the specific heat $\Delta B\, dE/dB$ in natural units of Kelvin as a function of temperature, for selected FWHM Γ of **a** symmetric and **b** asymmetric DOS peak. The red dashed line is the naively expected $\Delta B\, dE/dB = 4.8T$ dependence. The inset show the double-peak structure splitting in the calculated specific heat as a function of DOS peak width Γ.

Supplementary Note 11: Peak position in MCE and specific heat for symmetric and asymmetric DOS

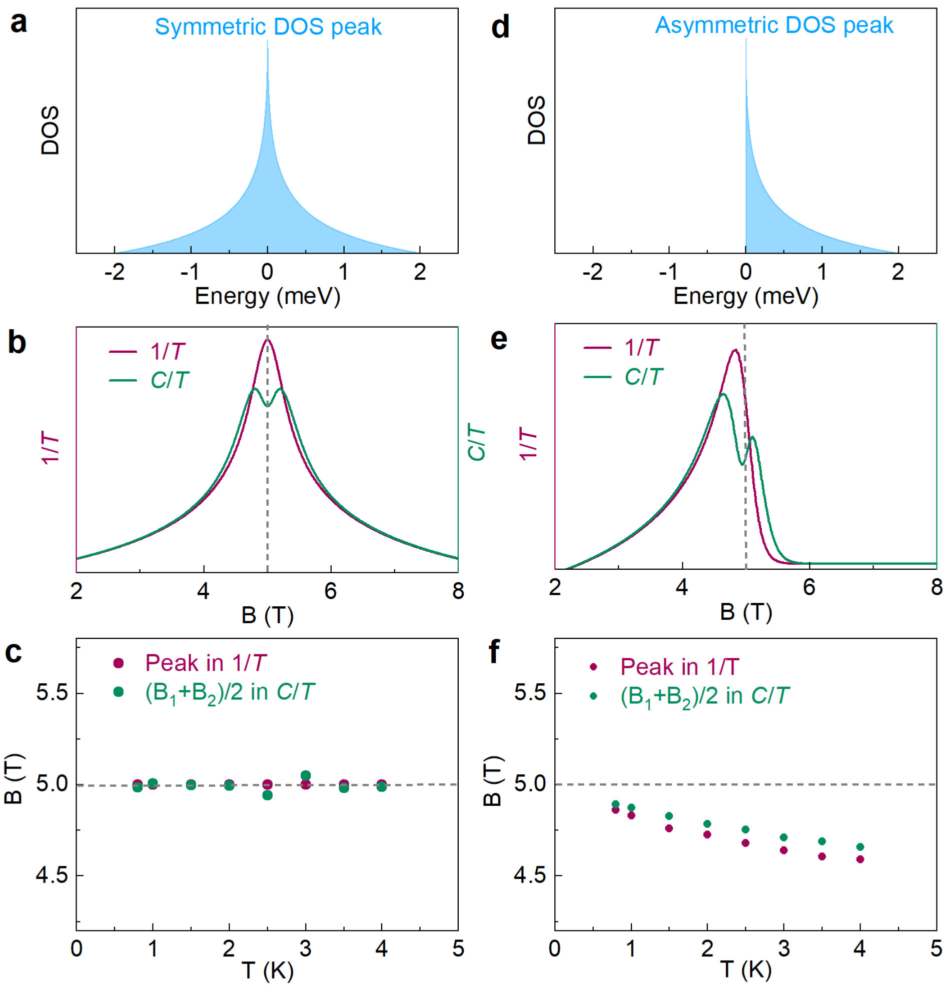

Supplementary Figure 10. **a - b** Symmetric and asymmetric DOS peak used to calculate the MCE ($1/T$) and the specific heat (C/T). In each case the DOS peak crosses E_F at $B = 5\,\mathrm{T}$. **c - d** Calculated $1/T$ and C/T curve at $T = 1\,\mathrm{K}$ for symmetric and asymmetric DOS peaks, respectively. **d - e** Position of the peak in $1/T$ and the center of double-peak structure ($(B_1+B_2)/2$) at different temperature for symmetric and asymmetric DOS, respectively.

In a simple picture, with a symmetric DOS peak, the peak position of MCE ($1/T$) lies exactly in the middle of the double peaks in C_el/T (see Supplementary Fig. 10a-c). However, when the DOS peak is asymmetric, the peak position of MCE ($1/T$) deviates from the center of double peaks in C_el/T (see Supplementary Fig. 10d-f). For an asymmetric DOS, both

features occur at magnetic fields slight below the $B = 5\,\mathrm{T}$ crossing of the Fermi energy.

Supplementary Note 12: Double-peak structure of specific heat near the Lifshitz transition

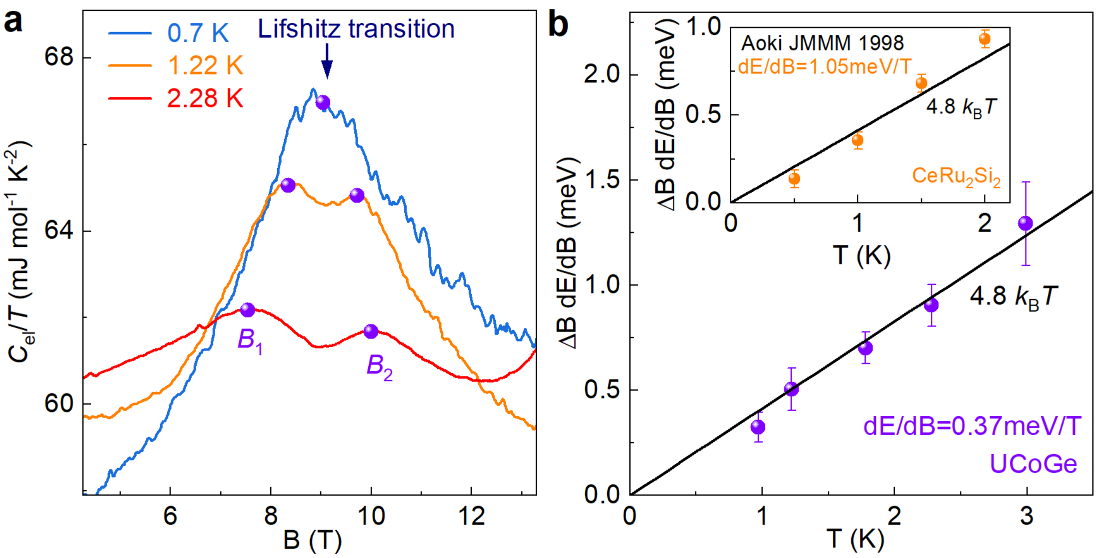

Supplementary Figure 11. **a** Fermionic quasiparticle specific heat divided by temperature C_{el}/T in UCoGe as a function of magnetic field in the vicinity of Lifshitz transition showing a double-peak structure splitting. Note that the symmetrical nature of the peaks, reflects the cusp like "singularity" in the DOS close to the Lifshitz transition, compared to the asymmetric nature of the Landau level DOS "singularity" in graphite. **b** Temperature dependence of the energy $\Delta B\, dE/dB$ through which "singularity" moves in UCoGe. The inset shows the same plot for ΔB extracted from the published C_{el}/T data of Aoki *et al.* [24] on the heavy fermion compound $CeRu_2Si_2$.

In addition to the quantum oscillation in graphite reported here, a double-peak structure (double peak) is occasionally observed in quasi-particle specific heat C_{el}/T near the Lifshitz transition, where a cusp like "singularity" occurs in the DOS [25, 26]. As an example, in Supplementary Fig. 11a we show C_{el}/T of UCoGe in the vicinity of Lifshitz transition. UCoGe is ferromagnetic superconductor that exhibiting a Lifshitz transition at B_c=9.5 T [27]. The field sweep C_{el}/T near the Lifshiz transition of UCoGe exhibits same double-peak structure observed in the specific heat of graphite. A similar behaviour is also observed in the Lifshitz transition of $CeRu_2Si_2$ at B_c=7.7 T [24]. The dE/dB for both $CeRu_2Si_2$ and UCoGe is estimated from the relation $\Delta B(dE/dB) = 4.8 k_B T$, as shown in Supplementary Fig. 11b.

Supplementary Note 13: Fitting of C/T near the Lifshitz transition of $CeRu_2Si_2$

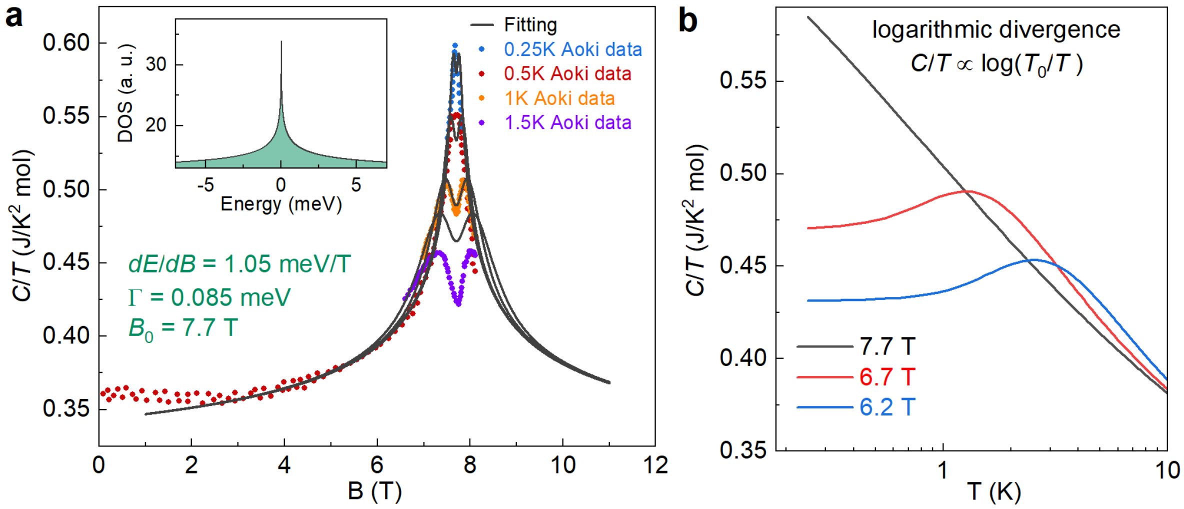

Supplementary Figure 12. **a** Data points are the digitized C/T versus B data near the Lifshitz transition of $CeRu_2Si_2$ from ref [24]. The solid black lines is the calculated behavior of C/T, for a *single* symmetric DOS peak passing through the Fermi energy, using the parameters indicated on the figure. The inset shows the *single* symmetric DOS peak used in the calculation which nicely reproduces the double-peak structure in C/T versus B lending further support to our model. **b** Semi-log plot of calculated C/T versus T using a *single* symmetric DOS peak at constant magnetic fields. At critical field B=7.7 T, C/T versus T shows a logarithmic divergence.

Supplementary Fig. 12a shows the digitized C/T data points near the Lifshitz transition of $CeRu_2Si_2$ from Aoki et al. [24]. It is predicted theoretically that the DOS peak in the subband edge of $CeRu_2Si_2$ is cusp-like ($D(E) \propto E^{-0.5}$) [26]. We therefore use a symmetric single cusp-like DOS peak (see inset of Supplementary Fig. 12a) to calculate the C/T, as shown by black solid lines in Supplementary Fig. 12a, using the parameters indicated on the figure. The fits are in an excellent agreement with the experimental data up to 1 K, but start to deviate in overall amplitude when the temperature is higher than 1 K. The discrepancy occurs because the temperature-dependent of the shape of DOS peak is not taken into account in our simple model.

The excellent agreement between the calculated C/T using a single symmetric DOS peak and the experimental results clearly demonstrates that only one subband (DOS peak)

passes through the Fermi energy at the Lifshitz transition of $CeRu_2Si_2$. Moreover, the MCE curve of $CeRu_2Si_2$ only exhibit a single-peak feature [24], which is fully consistent with our model. The simultaneous occurrence of single- and double-peak features in MCE and specific heat are smoking gun thermodynamic signatures of a single DOS peak crossing the Fermi level. Moreover, the magneto-resistance, Hall resistivity and thermopower measurements on $CeRu_2Si_2$ also suggest the same scenario [28][29], that is, a single spin subband crosses the Fermi energy at the Lifshitz transition of $CeRu_2Si_2$.

In heavy fermion system, it is known that a 'logarithmic divergence' feature in temperature sweep of specific heat ($C/T \propto \log{(T_0/T)}$) is a signature of non-Fermi Liquid behaviour [30]. Here, it is interesting to note that such logarithmic divergence feature can also be observed in C/T versus T for a fermionic singularity in DOS that passes over the Fermi energy. In Supplementary Fig. 12b, we show the semi-log plot of calculated C/T as a function of temperature using *single* symmetric DOS peak at constant magnetic fields. If the system has a logarithmic divergence behaviour, the temperature-sweep of C/T exhibits a straight line in the semi-log plot. As seen in Supplementary Fig. 12b, C/T versus T exhibits as a straight line at the critical field $B = 7.7\,\mathrm{T}$. Therefore, the logarithmic divergence in C/T is not only due to an existence of a canonical quantum critical point, but can also be attributed to the formation of a fermionic DOS singularity.

Supplementary Note 14: Advantage of extracting effective g-factor from double-peak structure in C_{el}/T

In most cases of quantum oscillations, the exact shape of the DOS is unknown which makes it difficult fit the data in order to extract the g-factor, dE/dB *etc*. Typically the $1/B$ periodicity of the oscillations are used to exactly calipers the Fermi surface.

As described in more detail below, to extract the g-factor using techniques such as SdHs, dHvA, MCE *etc*, one has to know the Landau index (orbital quantum number) for each peak, and the system dependent Fermi energy shift. This type of difficulty is discussed at some length in the classic book of D. Shoenberg ≪ Magnetic Oscillations in Metals≫ [31].

We stress once more, that crucially, the double-peak feature observed in specific heat (or eventually thermal conductance), when a single DOS peak crosses the Fermi energy, allows us to estimate the g-factor, without having to make any assumptions concerning the Landau index or Fermi energy shift.

Supplementary Note 14.1: Extraction of g-factor from position of spin-up and spin-down peaks in SdH, dHvA, MCE

Quantum oscillations, which are driven by the magnetic field dependent Landau level degeneracy, exactly caliper the Fermi surface, independently of the values of the cyclotron, or Zeeman energies. They are therefore, by definition, not well adapted to determine these quantities. In SdH, dHvA MCE measurements a spin split Landau level crossing the Fermi energy gives rise to two peaks. We can define a magnetic field splitting $\Delta B = B^+ - B^-$, where B^+ and B^- are the magnetic field position of spin up and spin down features. To a reasonable approximation, the g-factor can be estimated using,

$$\Delta B = \frac{g^* \mu_B B_m}{(N + 1/2)\hbar e/m^* - S_F}, \tag{19}$$

where $B_m = (B^+ + B^-)/2$ is the mean field position for B^+ and B^-, N is the Landau index, m^* is the effective mass, S_F is the slope of the Fermi energy in the Nth Landau level. To obtain g-factor from Supplementary Eq. (19), the Landau index and Fermi energy shift for the relevant spin-split Landau level are required, which are generally difficult to identify in a new system.

Supplementary Note 14.2: Extraction of g-factor from double peak in C/T using coincidence method

In specific heat (eventually thermal transport) measurements, a spin split Landau level crossing the Fermi energy should gives rise to four peaks *i.e.* two independent double-peak structure. However, under special conditions referred to as coincidence, two of the peaks occur at exactly the same magnetic field. Supplementary Fig. 13a shows the magnetic field position of the quadruple-peak structure $B^{+}_{1,2}$, $B^{-}_{1,2}$ as a function of temperature for a spin up and a spin down levels with same Landau index N. At a critical temperature T_c, the B^{-}_{2} peak and the B^{+}_{1} peak occur at the same magnetic field B_c. At this temperature, the $4.8k_BT_c$ splitting of $-x^2dF/dx$ has the correct value, so that the spin-up and spin-down DOS peaks are simultaneously located at one of the two maxima of $-x^2dF/dx$, as schematically illustrated in Supplementary Fig. 13b.

At the experimentally determined $T = T_c$, the energy gap between spin-up and spin-down levels is equal to the splitting of the maxima in $-x^2dF/dx$, therefore, we have,

$$g^*\mu_B B_c = 4.8k_B T_c, \tag{20}$$

Therefore, the coincidence condition (experimentally determined B_c and T_c) allows us to extract the g-factor without knowing the Landau index, or making any assumptions concerning the Fermi energy shift.

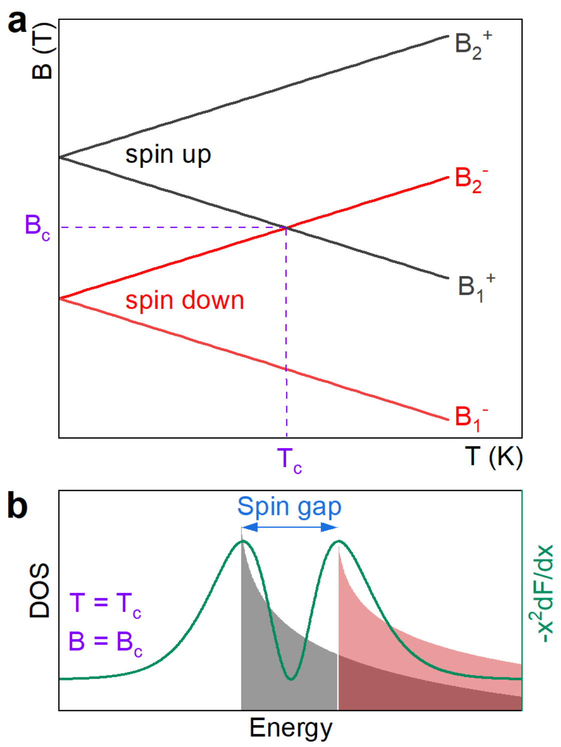

Supplementary Figure 13. **a** Schematic to show the magnetic field position of the two independent double-peak structures $B^+_{1,2}$, $B^-_{1,2}$ as a function of temperature when spin up/spin down levels with the same orbital quantum number are in the vicinity of the Fermi energy. **b** Schematic showing the coincidence condition at $T = T_c$ when the spin-up/down spin split levels simultaneously lie at the centre of one of the two maxima in $-x^2 dF/dx$

.

[1] T. Champel and V. Mineev, de haas–van alphen effect in two-and quasi-two-dimensional metals and superconductors, *Philos. Mag. B* **81**, 55 (2001).

[2] S. C. Riggs, O. Vafek, J. Kemper, J. Betts, A. Migliori, F. Balakirev, W. Hardy, R. Liang, D. Bonn, and G. Boebinger, Heat capacity through the magnetic-field-induced resistive transition in an underdoped high-temperature superconductor, *Nat. Phys.* **7**, 332 (2011).

[3] V. Bondarenko, S. Uji, T. Terashima, C. Terakura, S. Tanaka, and S. Maki, First observations of the heat capacity quantum oscillations in the organic superconductor (BEDT-TTF)$_2$Cu(NCS)$_2$, *Synth. Met.* **120**, 1039 (2001).

[4] J. Kačmarčík, I. Vinograd, B. Michon, A. Rydh, A. Demuer, R. Zhou, H. Mayaffre, R. Liang, W. N. Hardy, D. A. Bonn, N. Doiron-Leyraud, L. Taillefer, M.-H. Julien, C. Marcenat, and T. Klein, Unusual Interplay between Superconductivity and Field-Induced Charge Order in $YBa_2Cu_3O_y$, *Phys. Rev. Lett.* **121**, 167002 (2018).

[5] B. Michon, C. Girod, S. Badoux, J. Kačmarčík, Q. Ma, M. Dragomir, H. Dabkowska, B. Gaulin, J.-S. Zhou, S. Pyon, *et al.*, Thermodynamic signatures of quantum criticality in cuprate superconductors, *Nature* **567**, 218 (2019).

[6] P. F. Sullivan and G. Seidel, Steady-state, AC-temperature calorimetry, *Phys. Rev.* **173**, 679 (1968).

[7] N. B. Brandt, S. M. Chudinov, and Y. G. Ponomarev, *Semimetals: 1. Graphite and its compounds* (Elsevier, 2012).

[8] C. Kittel and P. McEuen, *Introduction to solid state physics*, Vol. 8 (Wiley New York, 1996).

[9] C. Marcenat, T. Klein, D. LeBoeuf, A. Jaoui, G. Seyfarth, J. Kačmarčík, Y. Kohama, H. Cercellier, H. Aubin, K. Behnia, and B. Fauqué, Wide critical fluctuations of the field-induced phase transition in graphite, *Phys. Rev. Lett.* **126**, 106801 (2021).

[10] J. M. Schneider, B. A. Piot, I. Sheikin, and D. K. Maude, Using the de Haas-van Alphen effect to map out the closed three-dimensional fermi surface of natural graphite, *Phys. Rev. Lett.* **108**, 117401 (2012).

[11] J. C. Slonczewski and P. R. Weiss, Band structure of graphite, *Phys. Rev.* **109**, 272 (1958).

[12] J. W. McClure, Theory of diamagnetism of graphite, *Phys. Rev.* **119**, 606 (1960).

[13] S. J. Williamson, S. Foner, and M. S. Dresselhaus, de Haas-van Alphen effect in pyrolytic and

single-crystal graphite, *Phys. Rev.* **140**, A1429 (1965).

[14] J. M. Schneider, M. Orlita, M. Potemski, and D. K. Maude, Consistent interpretation of the low-temperature magnetotransport in graphite using the Slonczewski-Weiss-Mcclure 3D band-structure calculations, *Phys. Rev. Lett.* **102**, 166403 (2009).

[15] K. Nakao, Landau level structure and magnetic breakthrough in graphite, *J. Phys. Soc. Japan* **40**, 761 (1976).

[16] J. M. Schneider, N. A. Goncharuk, P. Vašek, P. Svoboda, Z. Výborný, L. Smrčka, M. Orlita, M. Potemski, and D. K. Maude, Using magnetotransport to determine the spin splitting in graphite, *Phys. Rev. B* **81**, 195204 (2010).

[17] N. Miura, *Physics of semiconductors in high magnetic fields*, Vol. 15 (OUP Oxford, 2007).

[18] J. M. Schneider, *Electronic properties of graphite*, Ph.D. thesis, Grenoble; Université Joseph-Fourier-Grenoble I (2010), https://www.theses.fr/2010GRENY047.

[19] S. Blundell, *Magnetism in condensed matter* (Oxford University Press, 2003).

[20] K. Behnia, *Fundamentals of thermoelectricity* (Oxford University Press, 2015).

[21] H.-J. Hoffmann, From heat to entropy, *Materials Science & Engineering Technology* **51**, 1191 (2020).

[22] J. A. Woollam, Graphite carrier locations and quantum transport to 10T (100kG), *Phys. Rev. B* **3**, 1148 (1971).

[23] Z. Zhu, H. Yang, B. Fauque, Y. Kopelevich, and K. Behnia, Nernst effect and dimensionality in the quantum limit, *Nat. Phys.* **6**, 26 (2010).

[24] Y. Aoki, T. Matsuda, H. Sugawara, H. Sato, H. Ohkuni, R. Settai, E. Yamamoto, Y. Haga, A. Andreev, V. Sechovsky, *et al.*, Thermal properties of metamagnetic transition in heavy-fermion systems, *J. Magn. Magn. Mater.* **177**, 271 (1998).

[25] R. Mori, P. B. Marshall, K. Ahadi, J. D. Denlinger, S. Stemmer, and A. Lanzara, Controlling a Van Hove singularity and Fermi surface topology at a complex oxide heterostructure interface, *Nat. Commun.* **10**, 1 (2019).

[26] K. Miyake and H. Ikeda, True meaning of "localized" f-electrons measured by dHvA experiments in Ce-based heavy fermion metals, *J. Phys. Soc. Japan* **75**, 033704 (2006).

[27] G. Bastien, A. Gourgout, D. Aoki, A. Pourret, I. Sheikin, G. Seyfarth, J. Flouquet, and G. Knebel, Lifshitz transitions in the ferromagnetic superconductor UCoGe, *Phys. Rev. Lett.* **117**, 206401 (2016).

[28] R. Daou, C. Bergemann, and S. R. Julian, Continuous evolution of the fermi surface of ceru$_2$si$_2$ across the metamagnetic transition, *Phys. Rev. Lett.* **96**, 026401 (2006).

[29] H. Pfau, R. Daou, M. Brando, and F. Steglich, Thermoelectric transport across the metamagnetic transition of $CeRu_2Si_2$, *Phys. Rev. B* **85**, 035127 (2012).

[30] R. Küchler, P. Gegenwart, K. Heuser, E.-W. Scheidt, G. R. Stewart, and F. Steglich, Grüneisen Ratio Divergence at the Quantum Critical Point in $CeCu_{6-x}Ag_x$, *Phys. Rev. Lett.* **93**, 096402 (2004).

[31] D. Shoenberg, *Magnetic oscillations in metals* (Cambridge university press, 2009).
